# Initial Public Health Response and Interim Clinical Guidance for the 2019 Novel Coronavirus Outbreak — United States, December 31, 2019–February 4, 2020

**DOI:** 10.15585/mmwr.mm6905e1

**Published:** 2020-02-07

**Authors:** Anita Patel, Daniel B. Jernigan, 2019-nCoV CDC Response Team, 2019-nCoV CDC Response Team, Fatuma Abdirizak, Glen Abedi, Sharad Aggarwal, Denise Albina, Elizabeth Allen, Lauren Andersen, Jade Anderson, Megan Anderson, Tara Anderson, Kayla Anderson, Ana Cecilia Bardossy, Vaughn Barry, Karlyn Beer, Michael Bell, Sherri Berger, Joseph Bertulfo, Holly Biggs, Jennifer Bornemann, Josh Bornstein, Willie Bower, Joseph Bresee, Clive Brown, Alicia Budd, Jennifer Buigut, Stephen Burke, Rachel Burke, Erin Burns, Jay Butler, Russell Cantrell, Cristina Cardemil, Jordan Cates, Marty Cetron, Kevin Chatham-Stephens, Kevin Chatham-Stevens, Nora Chea, Bryan Christensen, Victoria Chu, Kevin Clarke, Angela Cleveland, Nicole Cohen, Max Cohen, Amanda Cohn, Jennifer Collins, Rebecca Dahl, Walter Daley, Vishal Dasari, Elizabeth Davlantes, Patrick Dawson, Lisa Delaney, Matthew Donahue, Chad Dowell, Jonathan Dyal, William Edens, Rachel Eidex, Lauren Epstein, Mary Evans, Ryan Fagan, Kevin Farris, Leora Feldstein, LeAnne Fox, Mark Frank, Brandi Freeman, Alicia Fry, James Fuller, Romeo Galang, Sue Gerber, Runa Gokhale, Sue Goldstein, Sue Gorman, William Gregg, William Greim, Steven Grube, Aron Hall, Amber Haynes, Sherrasa Hill, Jennifer Hornsby-Myers, Jennifer Hunter, Christopher Ionta, Cheryl Isenhour, Max Jacobs, Kara Jacobs Slifka, Daniel Jernigan, Michael Jhung, Jamie Jones-Wormley, Anita Kambhampati, Shifaq Kamili, Pamela Kennedy, Charlotte Kent, Marie Killerby, Lindsay Kim, Hannah Kirking, Lisa Koonin, Ram Koppaka, Christine Kosmos, David Kuhar, Wendi Kuhnert-Tallman, Stephanie Kujawski, Archana Kumar, Alexander Landon, Leslie Lee, Jessica Leung, Stephen Lindstrom, Ruth Link-Gelles, Joana Lively, Xiaoyan Lu, Brian Lynch, Lakshmi Malapati, Samantha Mandel, Brian Manns, Nina Marano, Mariel Marlow, Barbara Marston, Nancy McClung, Liz McClure, Emily McDonald, Oliva McGovern, Nancy Messonnier, Claire Midgley, Danielle Moulia, Janna Murray, Kate Noelte, Michelle Noonan-Smith, Kristen Nordlund, Emily Norton, Sara Oliver, Mark Pallansch, Umesh Parashar, Anita Patel, Manisha Patel, Kristen Pettrone, Taran Pierce, Harald Pietz, Satish Pillai, Lewis Radonovich, Sarah Reagan-Steiner, Amy Reel, Heather Reese, Brian Rha, Philip Ricks, Melissa Rolfes, Shahrokh Roohi, Lauren Roper, Lisa Rotz, Janell Routh, Senthil Kumar Sakthivel, Luisa Sarmiento, Jessica Schindelar, Eileen Schneider, Anne Schuchat, Sarah Scott, Varun Shetty, Caitlin Shockey, Jill Shugart, Mark Stenger, Matthew Stuckey, Brittany Sunshine, Tamara Sykes, Jonathan Trapp, Timothy Uyeki, Grace Vahey, Amy Valderrama, Julie Villanueva, Tunicia Walker, Megan Wallace, Lijuan Wang, John Watson, Angie Weber, Cindy Weinbaum, William Weldon, Caroline Westnedge, Brett Whitaker, Michael Whitaker, Alcia Williams, Holly Williams, Ian Willams, Karen Wong, Amy Xie, Anna Yousef

**Affiliations:** 1 Incident Manager, 2019-nCoV CDC Response, CDC.; National Center for Immunization and Respiratory Diseases, CDC; National Center for Immunization and Respiratory Diseases, CDC; National Center for Immunization and Respiratory Diseases, CDC; National Center for Emerging and Zoonotic Infectious Diseases, CDC; National Center for Emerging and Zoonotic Infectious Diseases, CDC; National Center for Emerging and Zoonotic Infectious Diseases, CDC; Center for Preparedness and Response, CDC; Center for Preparedness and Response, CDC; Center for State, Tribal, Local and Territorial Support, CDC; National Center on Birth Defects and Developmental Disabilities, CDC; National Center for Emerging and Zoonotic Infectious Diseases, CDC; National Center for Injury Prevention and Control, CDC; National Center for Emerging and Zoonotic Infectious Diseases, CDC; National Center for Emerging and Zoonotic Infectious Diseases, CDC; Office of the Director, CDC; Office of the Director, CDC; National Center for Immunization and Respiratory Diseases, CDC; Office of the Director, CDC; Office of the Director, CDC; National Center for Emerging and Zoonotic Infectious Diseases, CDC; National Center for Immunization and Respiratory Diseases, CDC; National Center for Emerging and Zoonotic Infectious Diseases, CDC; National Center for Immunization and Respiratory Diseases, CDC; National Center for Emerging and Zoonotic Infectious Diseases, CDC; National Center for Immunization and Respiratory Diseases, CDC; National Center for Immunization and Respiratory Diseases, CDC; National Center for Immunization and Respiratory Diseases, CDC; Office of the Deputy Director of Infectious Disease, CDC; Center for State, Tribal, Local and Territorial Support, CDC; National Center for Immunization and Respiratory Diseases, CDC; National Center for Immunization and Respiratory Diseases, CDC; National Center for Emerging and Zoonotic Infectious Diseases, CDC; National Center on Birth Defects and Developmental Disabilities, CDC; National Center on Birth Defects and Developmental Disabilities, CDC; National Center for Emerging and Zoonotic Infectious Diseases, CDC; National Center for Emerging and Zoonotic Infectious Diseases, CDC; National Center for Immunization and Respiratory Diseases, CDC; Center for Global Health, CDC; National Center for Immunization and Respiratory Diseases, CDC; National Center for Emerging and Zoonotic Infectious Diseases, CDC; Center for State, Tribal, Local and Territorial Support, CDC; National Center for Immunization and Respiratory Diseases, CDC; National Center for Emerging and Zoonotic Infectious Diseases, CDC; Erin Conners, National Center for Emerging and Zoonotic Infectious Diseases, CDC; Aaron Curns, National Center for Immunization and Respiratory Diseases, CDC; National Center for Immunization and Respiratory Diseases, CDC; Center for Preparedness and Response, CDC; Center for State, Tribal, Local and Territorial Support, CDC; Center for State, Tribal, Local and Territorial Support, CDC; National Center for Emerging and Zoonotic Infectious Diseases, CDC; National Institute for Occupational Safety and Health, CDC; Center for State, Tribal, Local and Territorial Support, CDC; National Institute for Occupational Safety and Health, CDC; National Center for Immunization and Respiratory Diseases, CDC; National Center for Immunization and Respiratory Diseases, CDC; , National Center for Emerging and Zoonotic Infectious Diseases, CDC; National Center for Emerging and Zoonotic Infectious Diseases, CDC; National Center for Injury Prevention and Control, CDC; National Center for Emerging and Zoonotic Infectious Diseases, CDC; National Center for Immunization and Respiratory Diseases, CDC; National Center for Immunization and Respiratory Diseases, CDC; National Center for Immunization and Respiratory Diseases, CDC; Center for Preparedness and Response, CDC; National Center for Immunization and Respiratory Diseases, CDC; National Center for Immunization and Respiratory Diseases, CDC; Center for Global Health, CDC; National Center for Chronic Disease Prevention and Promotion, CDC; National Center for Immunization and Respiratory Diseases, CDC; National Center for Emerging and Zoonotic Infectious Diseases, CDC; National Center for Immunization and Respiratory Diseases, CDC; Center for Preparedness and Response, CDC; National Center for Immunization and Respiratory Diseases, CDC; National Center for Emerging and Zoonotic Infectious Diseases, CDC; Office of the Director, CDC; National Center for Immunization and Respiratory Diseases, CDC; National Center for Immunization and Respiratory Diseases, CDC; National Center for Immunization and Respiratory Diseases, CDC; National Institute for Occupational Safety and Health, CDC; , National Center for Emerging and Zoonotic Infectious Diseases, CDC; National Center for Immunization and Respiratory Diseases, CDC; National Center for Immunization and Respiratory Diseases, CDC; Center for State, Tribal, Local and Territorial Support, CDC; National Center for Emerging and Zoonotic Infectious Diseases, CDC; National Center for Immunization and Respiratory Diseases, CDC; National Center for Emerging and Zoonotic Infectious Diseases, CDC; Center for Preparedness and Response, CDC; National Center for Immunization and Respiratory Diseases, CDC; National Center for Immunization and Respiratory Diseases, CDC; National Center for Immunization and Respiratory Diseases, CDC; Center for Surveillance; Epidemiology and Laboratory Services, CDC; National Center for Immunization and Respiratory Diseases, CDC; National Center for Immunization and Respiratory Diseases, CDC; National Center for Immunization and Respiratory Diseases, CDC; National Center for Immunization and Respiratory Diseases, CDC; National Center for Immunization and Respiratory Diseases, CDC; Center for Preparedness and Response, CDC; National Center for Emerging and Zoonotic Infectious Diseases; CDC; Deputy Director for Infectious Diseases, CDC; National Center for Immunization and Respiratory Diseases; CDC; , National Center for Immunization and Respiratory Diseases, CDC; Office of the Director, CDC; National Center for Immunization and Respiratory Diseases, CDC; National Center for Immunization and Respiratory Diseases, CDC; National Center for Immunization and Respiratory Diseases, CDC; National Center for Immunization and Respiratory Diseases, CDC; National Center for Immunization and Respiratory Diseases, CDC; National Center for Immunization and Respiratory Diseases, CDC; National Center for Immunization and Respiratory Diseases, CDC; National Center for Immunization and Respiratory Diseases, CDC; National Center for Immunization and Respiratory Diseases, CDC; National Center for Immunization and Respiratory Diseases, CDC; National Center for Emerging and Zoonotic Infectious Diseases, CDC; National Center for Immunization and Respiratory Diseases, CDC; Center for Global Health, CDC; National Center for Immunization and Respiratory Diseases, CDC; Center for Global Health, CDC; National Center for Emerging and Zoonotic Infectious Diseases, CDC; National Center for Immunization and Respiratory Diseases, CDC; National Center for Immunization and Respiratory Diseases, CDC; National Center for Immunization and Respiratory Diseases, CDC; National Center for Immunization and Respiratory Diseases, CDC; National Center for Immunization and Respiratory Diseases, CDC; Center for Preparedness and Response, CDC; Office of the Director, CDC; National Center for Immunization and Respiratory Diseases, CDC; National Institute for Occupational Safety and Health, CDC; National Center for Immunization and Respiratory Diseases, CDC; National Center for Immunization and Respiratory Diseases, CDC; National Center for Immunization and Respiratory Diseases, CDC; National Center for Immunization and Respiratory Diseases, CDC; National Center for Immunization and Respiratory Diseases, CDC; National Center for Health Statistics, CDC; National Center for Emerging and Zoonotic Infectious Diseases, CDC; Center for Preparedness and Response, CDC; National Center for Emerging and Zoonotic Infectious Diseases, CDC; National Institute for Occupational Safety and Health, CDC; National Center for Emerging and Zoonotic Infectious Diseases, CDC; National Center for Immunization and Respiratory Diseases, CDC; National Center for Immunization and Respiratory Diseases, CDC; National Center for Immunization and Respiratory Diseases, CDC; Center for Global Health, CDC; National Center for Immunization and Respiratory Diseases, CDC; National Center for Emerging and Zoonotic Infectious Diseases, CDC; National Center for Immunization and Respiratory Diseases, CDC; National Center for Emerging and Zoonotic Infectious Diseases, CDC; National Center for Immunization and Respiratory Diseases, CDC; National Center for Immunization and Respiratory Diseases, CDC; National Institute for Occupational Safety and Health, CDC; National Center for Emerging and Zoonotic Infectious Diseases, CDC; National Center for Immunization and Respiratory Diseases, CDC; Office of the Director, CDC; Center for State, Tribal, Local and Territorial Support, CDC; Center for State, Tribal, Local and Territorial Support, CDC; National Center for Emerging and Zoonotic Infectious Diseases, CDC; National Institute for Occupational Safety and Health, CDC; National Center for HIV/AIDS, Viral Hepatitis, STD, and TB Prevention; CDC; National Center for Emerging and Zoonotic Infectious Diseases, CDC; National Center for Emerging and Zoonotic Infectious Diseases, CDC; Office of the Director, CDC; Office of the Director, CDC; National Center for Immunization and Respiratory Diseases, CDC; National Center for Emerging and Zoonotic Infectious Diseases, CDC; National Center for Emerging and Zoonotic Infectious Diseases, CDC; National Center for Emerging and Zoonotic Infectious Diseases, CDC; Center for Preparedness and Response, CDC; National Center for Immunization and Respiratory Diseases, CDC; National Center for Immunization and Respiratory Diseases, CDC; National Center for Immunization and Respiratory Diseases, CDC; National Institute for Occupational Safety and Health, CDC; National Center for Immunization and Respiratory Diseases, CDC; National Center for Immunization and Respiratory Diseases, CDC; National Center for Immunization and Respiratory Diseases, CDC; National Center for Immunization and Respiratory Diseases, CDC; National Center for Immunization and Respiratory Diseases, CDC; Office of the Director, CDC; Office of the Director, CDC; National Center for Emerging and Zoonotic Infectious Diseases, CDC; Center for Surveillance, Epidemiology and Laboratory Services, CDC; Center for State, Tribal, Local and Territorial Support, CDC; National Center for Immunization and Respiratory Diseases, CDC.

On December 31, 2019, Chinese health officials reported a cluster of cases of acute respiratory illness in persons associated with the Hunan seafood and animal market in the city of Wuhan, Hubei Province, in central China. On January 7, 2020, Chinese health officials confirmed that a novel coronavirus (2019-nCoV) was associated with this initial cluster (*1*). As of February 4, 2020, a total of 20,471 confirmed cases, including 2,788 (13.6%) with severe illness,* and 425 deaths (2.1%) had been reported by the National Health Commission of China (*2*). Cases have also been reported in 26 locations outside of mainland China, including documentation of some person-to-person transmission and one death (*2*). As of February 4, 11 cases had been reported in the United States. On January 30, the World Health Organization (WHO) Director-General declared that the 2019-nCoV outbreak constitutes a Public Health Emergency of International Concern.^†^ On January 31, the U.S. Department of Health and Human Services (HHS) Secretary declared a U.S. public health emergency to respond to 2019-nCoV.^§^ Also on January 31, the president of the United States signed a “Proclamation on Suspension of Entry as Immigrants and Nonimmigrants of Persons who Pose a Risk of Transmitting 2019 Novel Coronavirus,” which limits entry into the United States of persons who traveled to mainland China to U.S. citizens and lawful permanent residents and their families (*3*). CDC, multiple other federal agencies, state and local health departments, and other partners are implementing aggressive measures to slow transmission of 2019-nCoV in the United States (*4*,*5*). These measures require the identification of cases and their contacts in the United States and the appropriate assessment and care of travelers arriving from mainland China to the United States. These measures are being implemented in anticipation of additional 2019-nCoV cases in the United States. Although these measures might not prevent the eventual establishment of ongoing, widespread transmission of the virus in the United States, they are being implemented to 1) slow the spread of illness; 2) provide time to better prepare health care systems and the general public to be ready if widespread transmission with substantial associated illness occurs; and 3) better characterize 2019-nCoV infection to guide public health recommendations and the development of medical countermeasures including diagnostics, therapeutics, and vaccines. Public health authorities are monitoring the situation closely. As more is learned about this novel virus and this outbreak, CDC will rapidly incorporate new knowledge into guidance for action by CDC and state and local health departments.

Some coronaviruses, such as Middle East Respiratory Syndrome (MERS) and Severe Acute Respiratory Syndrome (SARS), are the result of human-animal interactions. Preliminary investigation of 2019-nCoV also suggests a zoonotic origin (*6*), but the exact origin has not yet been determined. Person-to-person spread is evident (*7*); however, how easily the virus is transmitted between persons is currently unclear. 2019-nCoV is similar to coronaviruses that cause MERS and SARS, which are transmitted mainly by respiratory droplets. Signs and symptoms of patients with confirmed 2019-nCoV infection include fever, cough, and shortness of breath (*8*). Based on the incubation period of illness from MERS and SARS coronaviruses, CDC believes that symptoms of 2019-nCoV infection occur within 2 to 14 days following infection. Preliminary information suggests that older adults and persons with underlying health conditions or compromised immune systems might be at higher risk for severe illness from this virus (*9*); however, many characteristics of this novel coronavirus and how it might affect individual persons and potentially vulnerable population subgroups, such as the elderly or those with chronic health conditions, remain unclear.

## Epidemiology of First U.S. Cases

On January 21, 2020, the first person in the United States with diagnosed 2019-nCoV infection was reported. As of February 4, a total of 293 persons from 36 states, the District of Columbia, and the U.S. Virgin Islands were under investigation based on current patient under investigation (PUI) definitions,^¶^ and also included those being evaluated because they are close contacts. Of these PUIs, 11 patients have confirmed 2019-nCoV infection using a real-time reverse transcription–polymerase chain reaction (RT-PCR) assay developed by CDC. These 11 cases were diagnosed in the following states: Arizona (one), California (six), Illinois (two), Massachusetts (one), and Washington (one) (Table). Nine cases were in travelers from Wuhan. Eight of these nine cases were identified as a result of patients seeking clinical care for symptoms and clinicians connecting with the appropriate public health systems. Two cases (one each in California and Illinois) occurred in close contacts of two confirmed cases and were diagnosed as part of routine monitoring of case contacts. All patients are being monitored closely for progressing illness. No deaths have been reported in the United States.

**TABLE:** Characteristics of initial 2019 novel coronavirus cases (N = 11) — United States, January 21–February 4, 2020

Case	State	Approximate age (yrs)	Sex	Place of exposure	Date laboratory confirmation announced
1	Washington	30s	M	Wuhan	1/21/2020
2	Illinois	60s	F	Wuhan	1/24/2020
3	Arizona	20s	M	Wuhan	1/26/2020
4	California	30s	M	Wuhan	1/27/2020
5	California	50s	M	Wuhan	1/27/2020
6	Illinois	60s	M	Household Illinois	1/30/2020
7	California	40s	M	Wuhan	1/31/2020
8	Massachusetts	20s	M	Wuhan	2/01/2020
9	California	50s	F	Wuhan	2/02/2020
10	California	50s	M	Wuhan	2/02/2020
11	California	50s	F	Household California	2/02/2020

**Abbreviations:** F = female; M = male.

## Public Health Response

CDC established a 2019-nCoV Incident Management Structure on January 7, 2020. On January 21, CDC activated its Emergency Operations Center to optimize coordination for domestic and international 2019-nCoV response efforts. To date, CDC has deployed teams to the U.S. jurisdictions with cases to assist with epidemiologic investigation and to work closely with state and local partners to identify and monitor close contacts and better understand the spectrum of illness, transmission, and virulence associated with this novel virus. Information learned from these investigations will help inform response actions. CDC has closely monitored the global impact of this virus with staff members positioned in CDC offices around the world, including mainland China, and in coordination with other countries and WHO. This coordination has included deploying CDC staff members to work with WHO and providing active support to CDC offices in affected countries. In addition, CDC in response to the escalating risks of travel from China has issued a series of Travelers’ Health Notices for both Wuhan and the rest of China regarding the 2019-nCoV outbreak. On January 27, CDC issued a Level 3 travel notice for travelers to avoid all nonessential travel to mainland China.**

U.S. quarantine stations, located at 18 major U.S. ports of entry, are part of a comprehensive regulatory system authorized under section 361 of the Public Health Service Act (42 U.S. Code Section 264), that limits the introduction of infectious diseases into the United States to prevent their spread. On January 17, consistent with existing communicable disease response protocols, CDC Quarantine staff members instituted enhanced entry screening of travelers on direct and connecting flights from Wuhan, China, arriving at three major U.S. airports: Los Angeles (LAX), New York City (JFK), and San Francisco (SFO),^††^ which then expanded to include travelers arriving in Atlanta (ATL) and Chicago (ORD). These five airports together receive approximately 85% of all air travelers from Wuhan, China, to the United States. U.S. Customs and Border Protection officers identified travelers arriving from Wuhan and referred them to CDC for health screening.^§§^ Any traveler from Wuhan with signs or symptoms of illness (e.g., fever, cough, or difficulty breathing) received a more comprehensive public health assessment performed by CDC public health and medical officers.^¶¶^ All travelers from Wuhan were also provided CDC’s Travel Health Alert Notice (T-HAN)*** that advised them to monitor their health for 14 days and described recommended actions to take if relevant symptoms develop. As of February 1, 2020, a total of 3,099 persons on 437 flights were screened; five symptomatic travelers were referred by CDC to local health care providers for further medical evaluation, and one of these persons tested positive for 2019-nCoV.

On January 24, 2020, travel bans began to be instituted by the Chinese government, resulting in restricted travel in and out of Hubei Province, including the city of Wuhan, and fewer travelers undergoing entry screening in the United States. In response to the escalating risks associated with travel from mainland China, on January 31, 2020, the Presidential Proclamation further refined the border health strategy to temporarily suspend entry, undergo additional screening, or possible quarantine for individuals that have visited China (excluding Hong Kong, Macau, and Taiwan) in the past 14 days. These enhanced entry screening efforts are taking place at 11 airports at which all air travelers from China are being directed.

## Laboratory and Diagnostic Support

Chinese health officials posted the full 2019-nCoV genome sequence on January 10, 2020, to inform the development of specific diagnostic tests for this emergent coronavirus (*1*). Within a week, CDC developed a Clinical Laboratory Improvement Amendments–approved real-time RT-PCR test that can diagnose 2019-nCoV respiratory samples from clinical specimens. On January 24, CDC publicly posted the assay protocol for this test (https://www.cdc.gov/coronavirus/2019-nCoV/lab/index.html). On January 4, 2020, the Food and Drug Administration issued an Emergency Use Authorization to enable emergency use of CDC’s 2019-nCoV Real-Time RT-PCR Diagnostic Panel. To date, this test has been limited to use at CDC laboratories. This authorization allows the use of the test at any CDC-qualified lab across the country. CDC is working closely with FDA and public health partners, including the American Public Health Laboratories, to rapidly share these tests domestically and internationally through CDC’s International Reagent Resource (https://www.internationalreagentresource.org/). In addition, CDC uploaded the genome of the virus from the first reported cases in the United States to GenBank, the National Institutes of Health genetic sequence database of publicly available DNA sequences (https://www.ncbi.nlm.nih.gov/genbank/). CDC also is growing the virus in cell culture, which is necessary for further studies, including for additional genetic characterization. Once isolated, the virus will be made available through BEI Resources (https://www.beiresources.org/) to assist research efforts.

## Clinical and Infection Control Guidance

Additional information about 2019-nCoV is needed to better understand transmission, disease severity, and risk to the general population. Although CDC and partners are actively learning about 2019-nCoV, initial CDC guidance is based on guidance for management and prevention of respiratory illnesses including influenza, MERS, and SARS. No vaccine or specific treatment for 2019-nCoV infection is currently available. At present, medical care for patients with 2019-nCoV is supportive.

On January 31, CDC published its third Health Advisory with interim guidance for clinicians and public health practitioners.^†††^ In addition, CDC issued a Clinical Action Alert through its Clinician Outreach and Communication Activity network on January 31.^§§§^ Interim guidance for health care professionals is available at https://www.cdc.gov/coronavirus/2019-nCoV/hcp/clinical-criteria.html. Health care providers should identify patients who might have been exposed and who have signs or symptoms related to 2019-nCoV infection, isolate these patients, and inform public health departments. This includes obtaining a detailed travel history for patients being evaluated with fever and lower respiratory tract illness. Criteria to guide evaluation and testing of PUIs for 2019-nCoV include 1) fever or signs or symptoms of lower respiratory tract illness (e.g., cough or shortness of breath) in any person, including health care workers, who has had close contact^¶¶¶^ with a patient with laboratory-confirmed 2019-nCoV infection within 14 days of symptom onset; 2) fever and signs or symptoms of lower respiratory tract illness (e.g., cough or shortness of breath) in any person with a history of travel from Hubei Province, China, within 14 days of symptom onset; or 3) fever and signs or symptoms of lower respiratory tract illness (e.g., cough or shortness of breath) requiring hospitalization in any person with a history of travel from mainland China within 14 days of symptom onset. Additional nonhospitalized PUIs may be tested based on consultation with state and local public health officials. Clinicians should evaluate PUIs for other possible causes of illness (e.g., influenza and respiratory syncytial virus) as clinically indicated.

CDC currently recommends a cautious approach to the examination of PUIs. These patients should be asked to wear a surgical mask as soon as they are identified, and directed to a separate area, if possible, separated by at least 6 ft (2 m) from other persons. Patients should be evaluated in a private room with the door closed, ideally an airborne infection isolation room, if available. Health care personnel entering the room should use standard precautions, contact precautions, airborne precautions, and eye protection (e.g., goggles or a face shield).

Clinicians should immediately notify the health care facility’s infection control personnel and local health department. The health department will determine whether the patient needs to be considered a PUI for 2019-nCoV and be tested for infection. If directed by the health department, to increase the likelihood of detecting 2019-nCoV infection, CDC recommends collecting and testing both upper and lower respiratory tract specimens.**** Additional specimen types (e.g., stool or urine) may be collected and stored. Specimens should be collected as soon as possible once a PUI is identified regardless of time since symptom onset.

For persons who might have 2019-nCoV infection and their close contacts, information and guidance on how to reduce the risk for transmitting and acquiring infection is available at https://www.cdc.gov/coronavirus/2019-ncov/hcp/guidance-prevent-spread.html. Close contacts should immediately call their health care providers if they develop symptoms. In addition, CDC is working closely with state and local health partners to develop and disseminate information to the public on general prevention of respiratory illness, including the 2019-nCoV. This includes everyday preventive actions such as washing your hands, covering your cough, and staying home when you are ill. Additional information and resources for this outbreak are available on the CDC website (https://www.cdc.gov/coronavirus/2019-ncov/index.html).

## Discussion

The 2019-nCoV has impacted multiple countries, caused severe illness, and sustained person-to-person transmission making it a concerning and serious public health threat. It is unclear how this virus will impact the U.S. over time. For the general population, who are unlikely to be exposed to this virus at the current time, the immediate health risk from 2019-nCoV is considered low. CDC, multiple other federal agencies, state and local health departments, and other partners are implementing aggressive measures to slow U.S. transmission of 2019-nCoV (*4*,*5*). These measures require the identification of cases and contacts in the United States and the effective management of the estimated 14,000 travelers arriving from mainland China to the United States each day (*3*). These measures are being implemented based on the assumption that there will be more U.S. 2019-nCoV cases occurring with potential chains of transmission, with the understanding that these measures might not prevent the eventual establishment of ongoing, widespread transmission of the virus in the United States.

It is important for public health agencies, health care providers, and the public to be aware of this new 2019-nCoV so that coordinated, timely, and effective actions can help prevent additional cases or poor health outcomes. The critical role that the U.S. health care system plays in halting or significantly slowing U.S. transmission of 2019-nCoV is already evident: eight of the first 11 U.S. cases were detected by clinicians collaborating with public health to test persons at risk. The early recognition of cases in the United States reduces transmission risk and increases understanding of the virus, including its transmission and severity, to inform national and global response actions.

2019-nCoV symptoms are similar to those of influenza (e.g., fever, cough, or sore throat), and the outbreak is occurring during a time of year when respiratory illnesses from influenza, respiratory syncytial virus, and other respiratory viruses are highly prevalent. To prevent influenza, all persons aged ≥6 months should receive an annual influenza vaccine, and vaccination is still available and effective in helping to prevent influenza (*10*). Reducing the number of persons in the United States with seasonal influenza will reduce possible confusion with 2019-nCoV infection and possible additional risk to patients with seasonal influenza. Public health authorities are monitoring the situation closely. As more is learned about this novel virus and this outbreak, CDC will rapidly incorporate new knowledge into guidance for action.

**Summary**

**What is already known about this topic?**

In December 2019, an outbreak of acute respiratory illness caused by a novel coronavirus (2019-nCoV) was detected in mainland China. Cases have been reported in 26 additional locations, including the United States.

**What is added by this report?**

Nine of the first 11 U.S. 2019-nCoV patients were exposed in Wuhan, China. CDC expects more U.S. cases.

**What are the implications for public health practice?**

CDC, multiple other federal agencies, state and local health departments, and other partners are implementing aggressive measures to substantially slow U.S. transmission of 2019-nCoV, including identification of U.S. cases and contacts and managing travelers arriving from mainland China to the United States. Interim guidance is available at https://www.cdc.gov/coronavirus/index.html and will be updated as more information becomes available.

## Footnotes

* Includes any of the following: dyspnea, respiratory rate >30 breaths per minute, hypoxemia, or chest x-ray with multilobar infiltrates or >50% progression of pulmonary infiltration within 24–48 hours per WHO. https://www.who.int/docs/default-source/coronaviruse/situation-reports/20200123-sitrep-3-2019-ncov.pdf.

† https://www.who.int/news-room/detail/30-01-2020-statement-on-the-second-meeting-of-the-international-health-regulations-(2005)-emergency-committee-regarding-the-outbreak-of-novel-coronavirus-(2019-ncov).

§ https://www.phe.gov/emergency/news/healthactions/phe/Pages/2019-nCoV.aspx.

¶ Criteria to guide evaluation and testing of patients under investigation for 2019-nCoV include 1) fever or signs or symptoms of lower respiratory tract illness (e.g., cough or shortness of breath) in any person, including a health care worker, who has had close contact with a patient with laboratory-confirmed 2019-nCoV infection within 14 days of symptom onset; 2) fever and signs or symptoms of lower respiratory tract illness (e.g., cough or shortness of breath) in any person with a history of travel from Hubei Province, China, within 14 days of symptom onset; or 3) fever and signs or symptoms of lower respiratory tract illness (e.g., cough or shortness of breath) requiring hospitalization in any person with a history of travel from mainland China within 14 days of symptom onset. More information is available at https://emergency.cdc.gov/han/han00427.asp and https://emergency.cdc.gov/han/han00426.asp.

** https://wwwnc.cdc.gov/travel/notices/warning/novel-coronavirus-china.

†† https://www.cdc.gov/media/releases/2020/p0117-coronavirus-screening.html.

§§ CDC’s initial health screening includes a measurement of each traveler’s temperature with a handheld noncontact thermometer, observation of these travelers for visible signs of respiratory illness (e.g., cough or difficulty breathing), and review of symptoms through a self-administered questionnaire.

¶¶ The more comprehensive public health assessment determines, based on the traveler’s illness and exposure, whether the traveler should be taken to a hospital for further medical evaluation and care, which might include testing for 2019-nCoV.

*** https://www.cdc.gov/coronavirus/2019-ncov/travelers/communication-resources.html.

††† https://emergency.cdc.gov/han/han00427.asp.

§§§ https://emergency.cdc.gov/coca/calls/2020/callinfo_013120.asp.

¶¶¶ Close contact is defined as 1) being within approximately 6 ft (2 m) of a 2019-nCoV patient for a prolonged period while not wearing recommended personal protective equipment (PPE) (e.g., gowns, gloves, National Institute for Occupational Safety and Health–certified disposable N95 respirator, and eye protection); close contact can occur while caring for, living with, visiting, or sharing a health care waiting area or room with a 2019-nCoV patient; or 2) having direct contact with infectious secretions of a 2019-nCoV patient (e.g., being coughed on) while not wearing recommended PPE.

**** https://www.cdc.gov/coronavirus/2019-nCoV/lab/guidelines-clinical-specimens.html.
