# *Erratum*: Vol. 69, No. 5

**DOI:** 10.15585/mmwr.mm6906a5

**Published:** 2020-02-14

**Authors:** 2019-nCoV CDC Response Team, Faruque Ahmed, Olivia Almendares, Ashwin Belludi, Isaac Benowitz, Chris Braden, Christina Carlson, Howard Chiou, Nakia Clemmons, Dave Daigle, Meghna Desai, Lindsey Duca, Marc Fischer, Isaac Ghinai, Carolyn Greene, Cheri Grigg, Ardath Grills, Katherina Grusich, Benjamin Hallowell, Connor Hoff, Jesica Jacobs, Bradley King, John MacArthur, Claire Mattison, Jason McDonald, Tristan McPherson, Alexander Millman, Shannon Novosad, Mary Pomeroy, Noreen Qualls, Maryan Reynolds, Heather Rhodes, Rajeev Sharma, Robert Simmonds, Rebekah Stewart, Rebecca Sunenshine, Mark Tenforde, Amra Uzicanin, Jennifer Verani, Florence Whitehill, Kathryn Wilson, Jonathan Wortham

**Affiliations:** National Center for Emerging and Zoonotic Infectious Diseases, CDC; National Center for Immunization and Respiratory Diseases, CDC; National Center for Immunization and Respiratory Diseases, CDC; National Center for Emerging and Zoonotic Infectious Diseases, CDC; National Center for Emerging and Zoonotic Infectious Diseases, CDC; Center for Global Health, CDC; Center for Surveillance, Epidemiology, and Laboratory Services, CDC; National Center for Immunization and Respiratory Diseases, CDC; Center for Global Health, CDC; Center for Global Health, CDC; National Center for Chronic Disease Prevention and Health Promotion, CDC; National Center for Emerging and Zoonotic Infectious Diseases, CDC; Center for Surveillance, Epidemiology, and Laboratory Services, CDC; National Center for Immunization and Respiratory Diseases, CDC; National Center for Emerging and Zoonotic Infectious Diseases, CDC; National Center for Emerging and Zoonotic Infectious Diseases, CDC; National Center for Immunization and Respiratory Diseases, CDC; National Center for Immunization and Respiratory Diseases, CDC; National Center for Emerging and Zoonotic Infectious Diseases, CDC; Center for Surveillance, Epidemiology, and Laboratory Services, CDC; National Institute for Occupational Safety and Health, CDC; Center for Global Health, CDC; National Center for Immunization and Respiratory Diseases, CDC; Center for Preparedness and Response, CDC; Center for Surveillance, Epidemiology, and Laboratory Services, CDC; National Center for Immunization and Respiratory Diseases, CDC; National Center for Emerging and Zoonotic Infectious Diseases, CDC; National Center for Emerging and Zoonotic Infectious Diseases, CDC; National Center for Emerging and Zoonotic Infectious Diseases, CDC; National Center for Emerging and Zoonotic Infectious Diseases, CDC; Center for Surveillance, Epidemiology, and Laboratory Services, CDC; Center for Global Health, CDC; Center for Global Health, CDC; National Center for HIV/AIDS, Viral Hepatitis, STD, and TB Prevention, CDC; Center for Preparedness and Response, CDC; National Center for Immunization and Respiratory Diseases, CDC; National Center for Emerging and Zoonotic Infectious Diseases, CDC; Center for Global Health, CDC; National Center on Birth Defects and Developmental Disabilities, CDC; National Center for Emerging and Zoonotic Infectious Diseases, CDC; National Center for HIV/AIDS, Viral Hepatitis, STD, and TB Prevention, CDC.

In the report “Initial Public Health Response and Interim Clinical Guidance for the 2019 Novel Coronavirus Outbreak — United States, December 31, 2019–February 4, 2020,” on page 142, the fourth sentence of the first paragraph under “Laboratory and Diagnostic Support” should have read “On **February** 4, 2020 the Food and Drug Administration issued an Emergency Use Authorization to enable emergency use of CDC’s 2019-nCoV Real-Time RT-PCR Diagnostic Panel.”

On page 142, the first sentence of the first paragraph in the second column should have read “CDC is working closely with FDA and public health partners, including the **Association of** Public Health Laboratories, to rapidly share these tests domestically and internationally through CDC’s International Reagent Resource (https://www.internationalreagentresource.org/).”

In addition, names of the members of the 2019-nCoV CDC Response Team were omitted. The names are included below.

